# Superficial Venous Thrombosis of the Brachial Vein With Extension Into the Subclavian and Internal Jugular Veins, Secondary to a Routine Blood Draw

**DOI:** 10.7759/cureus.38260

**Published:** 2023-04-28

**Authors:** Jacob Loesche, Samuel Brown, Melissa Myers

**Affiliations:** 1 Emergency Medicine, Brooke Army Medical Center, Fort Sam Houston, USA

**Keywords:** basilic vein thrombus, jugular vein thrombosis, upper extremity thrombosis, deep vein thrombosis (dvt), superficial thrombophlebitis

## Abstract

Superficial thrombophlebitis, also known as superficial venous thrombosis, is an inflammatory condition involving the veins just below the surface of the skin secondary to clotted blood within that vein. The majority of cases are self-limited or resolve with a short course of anti-inflammatory medications and the application of warm compresses. Due to the self-limited nature of this disease process, clinically significant complications have rarely been described but are being seen more often in recent literature. This case report discusses an instance of superficial thrombophlebitis that occurred secondary to a routine blood draw and progressed to potentially life-threatening deep vein thrombosis. This case highlights the need for physicians to be aware of the potential complications of superficial thrombophlebitis and the importance of delivering strict return precautions to every patient with this condition.

## Introduction

Superficial thrombophlebitis, also known as superficial venous thrombosis, is a thrombotic event leading to inflammation of the superficial vasculature. Most common in the lower extremities, this condition arises due to typical thrombotic risk factors such as immobilization, vascular trauma, or a hypercoagulable state. The literature is divided in regards to an actual incidence of the condition, but it has been shown to account for 5.4% of the adjusted population attributable risk for initial DVT or pulmonary embolism [1,2].

Deep vein thrombosis (DVT) similarly occurs primarily in the lower extremity, with less than 10% occurring in the upper extremity [3]. However, a 2020 cohort study found that DVTs in the upper extremity are increasing. In the early 2000s, 1% to 4% of all reported DVTs occurred in the upper extremities. Comparatively, 10% of all DVTS today occur in the upper extremities, more than doubling the incidence from 20 years prior [4]. This is likely due to the progression of endovascular medical technology and more frequent usage of central venous catheters (CVCs) and especially peripherally inserted central catheters, also known as PICC lines.

Here, we present a case of superficial thrombophlebitis secondary to a routine blood draw that extended to become a DVT of the upper extremity involving the subclavian and the internal jugular veins in a patient with no malignancy or hematological clotting disorder.

## Case presentation

A 70-year-old male with a reported past medical history of hypothyroidism, hypertension, and mild dementia presented to the emergency department with a chief complaint of right upper extremity redness and swelling. The patient’s wife reported that her husband had a routine blood draw performed with a butterfly needle three days ago and since then had developed redness and swelling in the area. At the time of presentation, the patient denied other symptoms including fevers, chills, chest pain, dyspnea, nausea, vomiting, or loss of consciousness. Upon examination, it was noted that the patient had a 3 cm area of cord-like firmness with overlying edema and erythema on his right upper extremity. This area was tender to palpation and was assumed to be a superficial vein in the antecubital fossa.

**Figure 1:**
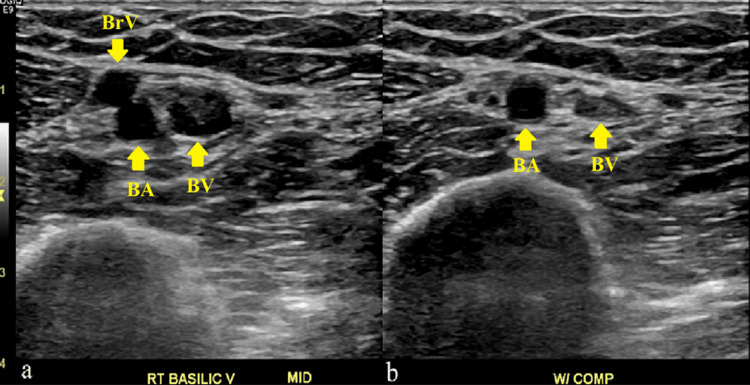
Non-compressible basilic vein (mid) suggesting superficial thrombosis Panel a (left) shows an ultrasound image of the basilic vein, the brachial artery, and another venous structure, likely a branch of the brachial vein. Panel b (right) shows the same ultrasound image, now compressed, with the basilic vein not fully compressed, indicating superficial thrombosis. Abbreviations: Right (RT), V (Vein), W/ COMP (With compression), BrV (Brachial vein), BA (Basilic artery), BV (Basilic vein)

**Figure 2:**
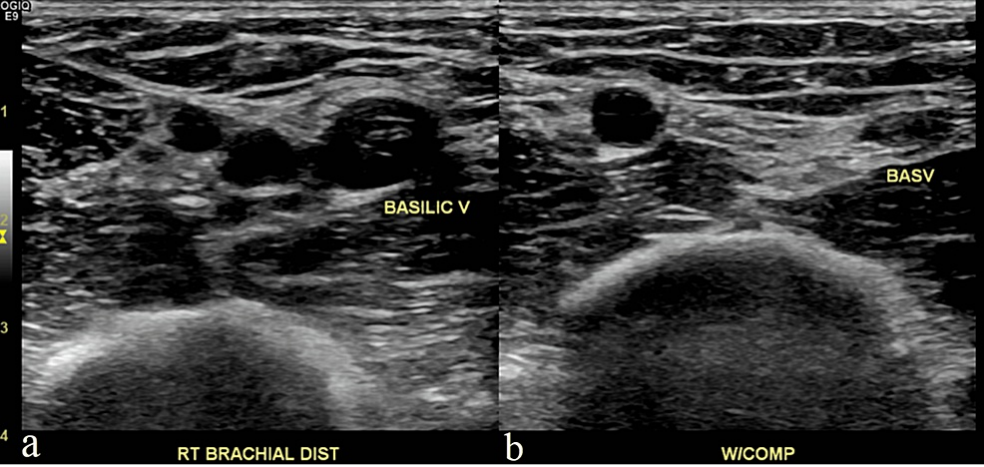
Non-compressible basilic vein (distal) suggesting superficial thrombosis Panel a (left) shows an ultrasound image of the basilic vein slightly more distally in comparison to Figure 1, the brachial artery, and another venous structure, likely a branch of the brachial vein. Panel b (right) shows the same ultrasound image, now compressed, with the basilic vein not fully compressed, indicating a superficial thrombosis. Abbreviations: RT (Right), Dist (Distal), V (Vein), W/COMP (With compression)

Bedside ultrasound (US) revealed a non-occlusive thrombus in the basilic vein underlying the area of erythema (Figure 1). A comprehensive Doppler ultrasound evaluation of the deep veins of the right upper extremity was ordered and confirmed the findings of the bedside sonographic examination. This study revealed a non-occlusive superficial thrombus running the length of the basilic vein (Figure 2). The internal jugular, subclavian, axillary, and brachial veins were found to be normal.

Due to the erythema surrounding the antecubital fossa, in conjunction with the US findings, the patient was diagnosed with superficial thrombophlebitis with overlying cellulitis. He was discharged with cephalexin and recommended alternating non-steroidal anti-inflammatory drugs and acetaminophen use as well as frequent warm compresses. The patient was discharged in good condition with plans to follow up with his primary care physician.

**Figure 3:**
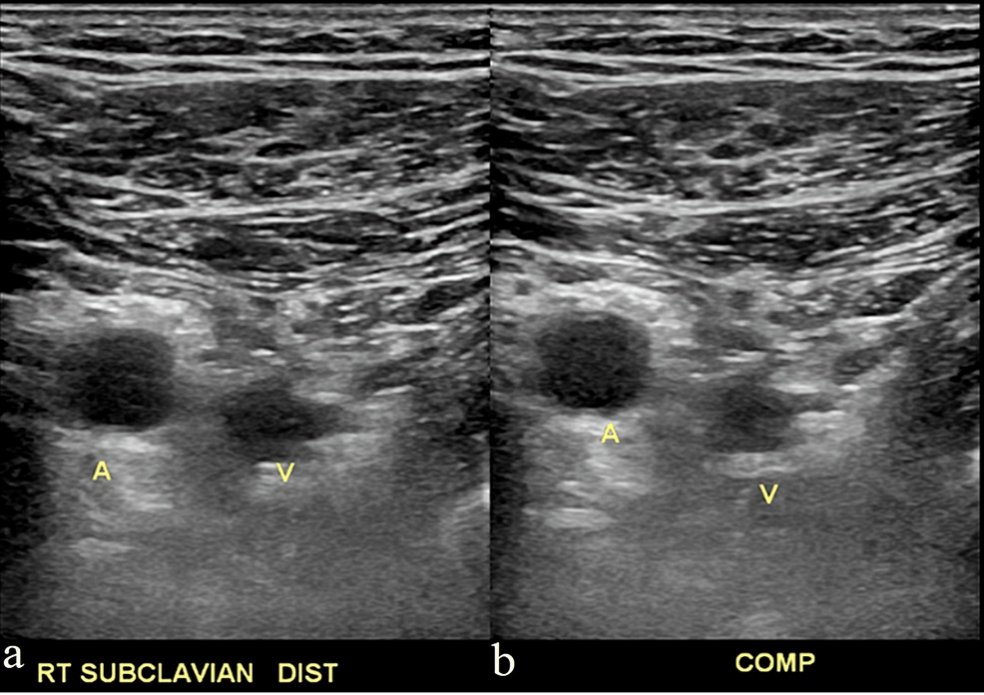
Non-compressible subclavian vein suggesting deep vein thrombosis Panel a (left) shows an ultrasound image of the subclavian artery and subclavian vein. Panel b (right) shows the same image, now with compression, and the subclavian vein not being compressed, indicating the presence of thrombosis. Abbreviations: A (Artery), V (Vein), RT (Right), Dist (Distal), COMP (With compression)

**Figure 4:**
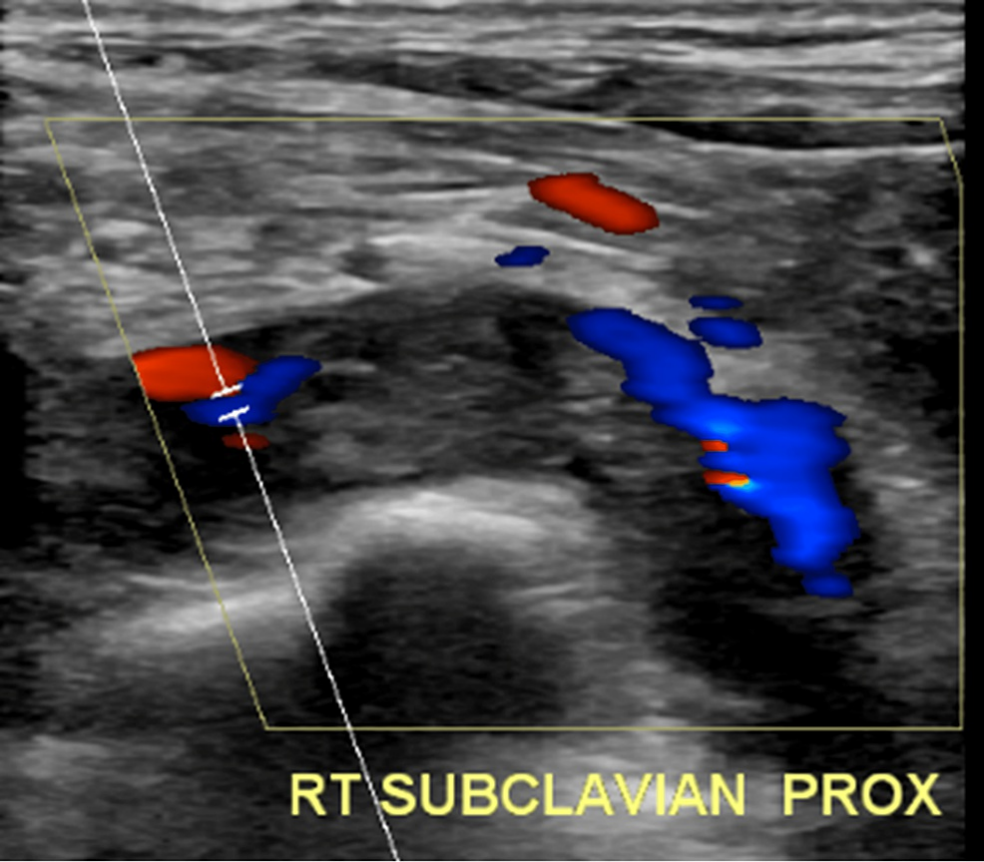
Doppler ultrasound evaluation showing persistent blood flow surrounding partially occlusive subclavian deep vein thrombosis The figure shows an ultrasound image with color Doppler enhancement to show the continuation of venous blood flow through the right subclavian vein around an area of increased echogenicity compared to the surrounding vessel. This is suggestive of a thrombosis that is partially occluding the vessel. Abbreviations: RT (Right), PROX (Proximal)

**Figure 5:**
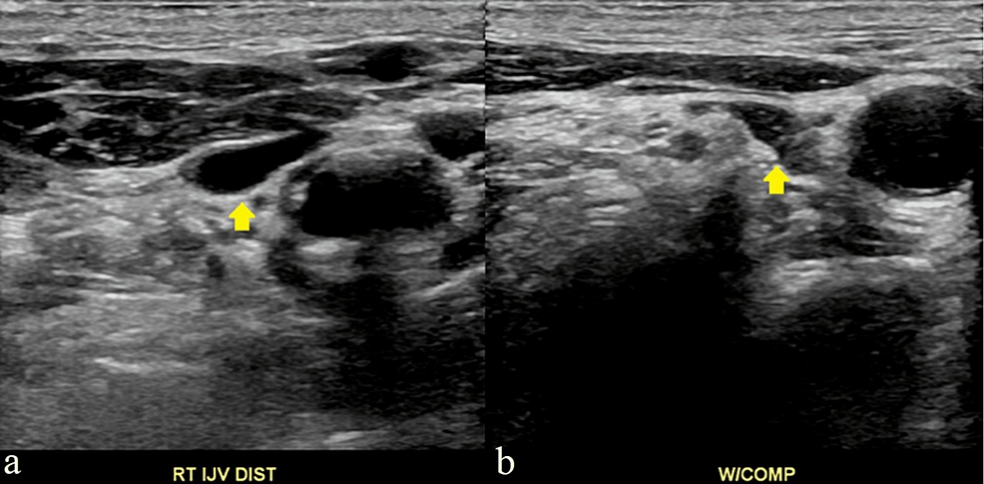
Non-compressible internal jugular vein suggesting deep vein thrombosis Panel a (left) shows an ultrasound image of the left internal jugular vein, indicated by an arrow, and the carotid artery. Panel b shows the same image, this time with compression, with the internal jugular only partially compressed indicating the presence of a deep vein thrombosis. Abbreviations: RT (Right), Dist (Distal), W/COMP (With compression), IJV (Internal jugular vein)

**Figure 6:**
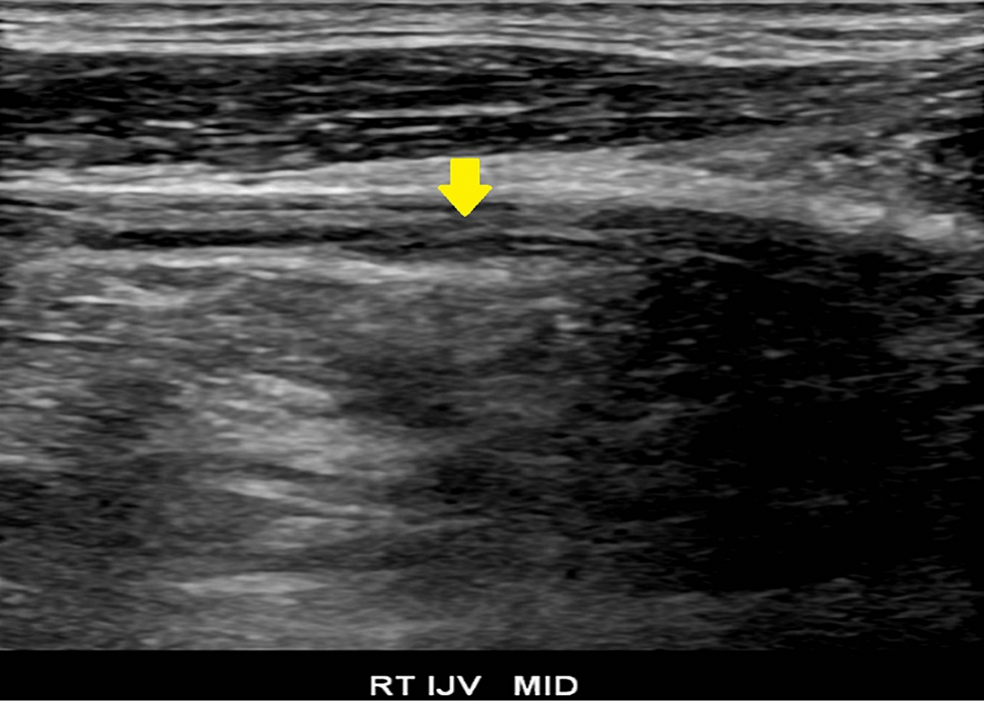
Longitudinal view of the right internal jugular vein with significant intraluminal clot burden Ultrasound image of the right internal jugular vein in a longitudinal orientation. The arrow indicates an area of increased echogenicity inside the vessel demonstrating an extended area of thrombosis. Abbreviations: RT (Right), IJV (Internal jugular vein)

**Figure 7:**
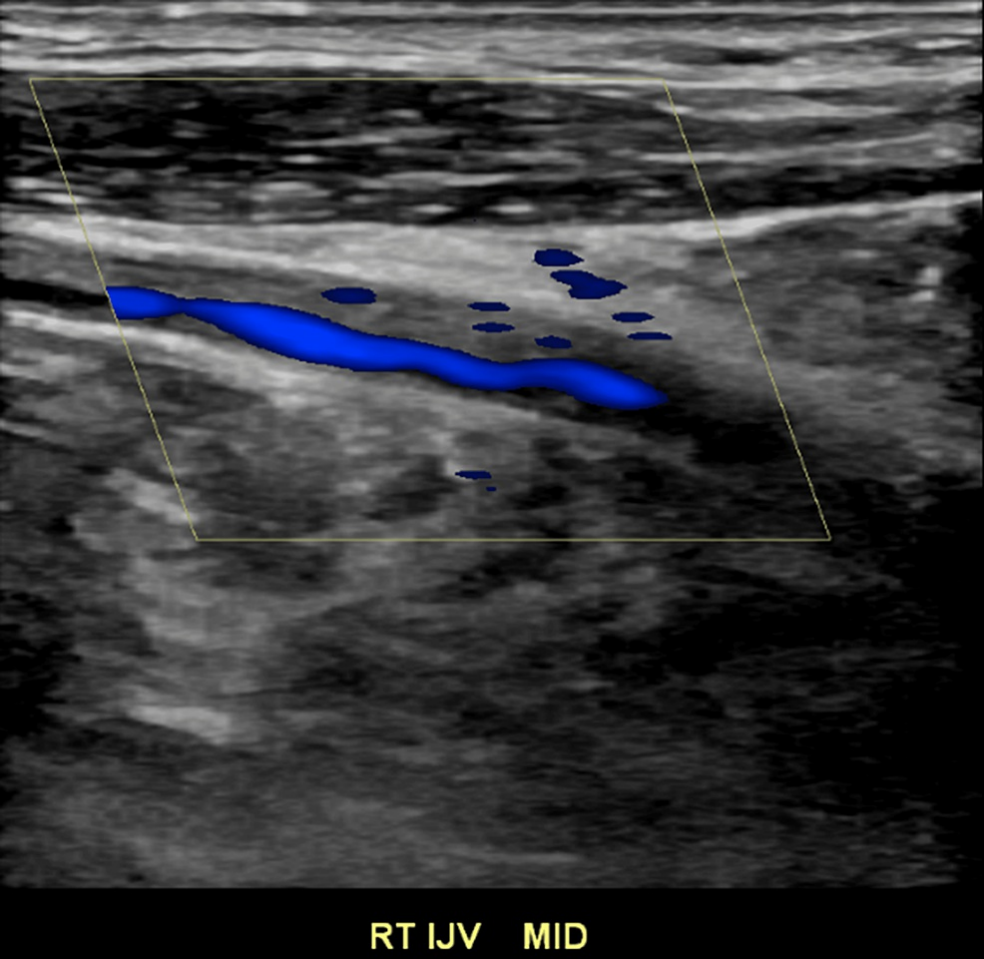
Doppler evaluation of the internal jugular vein showing partially occlusive deep vein thrombosis Ultrasound image with color Doppler enhancement of the right internal jugular vein with evidence of persistent blood flow around an area of increased echogenicity inside the vasculature indicating a partially occlusive deep vein thrombosis. Abbreviations: RT (Right), IJV (Internal jugular vein)

Nine days later, the patient returned to the emergency department with worsening pain with range of motion of his shoulder and swelling progressing up his arm and into his right axilla. Repeat comprehensive Doppler ultrasound examination revealed a partially occlusive thrombus within the right subclavian vein and right internal jugular veins with persistent occlusive thrombus within the right basilic vein (Figures 3-7). Due to the clot burden now extending into the deep subclavian and internal jugular veins, a heparin drip was started while the patient was in the emergency department and the patient was admitted to the internal medicine service. In discussion with the hospitalist, concern existed for underlying coagulopathy, however, among the patient's known medical history, family history, and calls made to outside hospital medical record departments, no history or evidence of a clotting disorder was found.

## Discussion

Our patient’s clot burden progressed from a superficial vein, the basilic vein, to involving the subclavian and internal jugular on the same side. The patient’s symptoms progressed similarly and expectedly. The patient originally presented with pain in the antecubital fossa, an indurated cord, and local erythema. These symptoms should prompt a provider to suspect the diagnosis of a superficial venous thrombosis. A retrospective study done in 2019 looking at the clinical features and etiology of upper extremity superficial venous thrombosis described similar presenting complaints with an indurated cord and erythema having an 85.7% positive predictive value for SVT [5]. Importantly, our patient was instructed to return should his symptoms progress, which ultimately led to his return and diagnosis of an upper extremity DVT.

The literature on upper extremity DVTs has increased over the last 15 years, as this condition is being found more frequently. As more information emerges regarding the inciting factors for these upper extremity clots, we see common causes of lower extremity DVTs being implicated, such as anatomic abnormalities causing venous congestion, inherited thrombophilia, and acquired hypercoagulable states such as malignancy and pregnancy. However, we also see endovascular provocation from the peripherally inserted central catheter (PICC) lines, CVCs, pacemaker/implantable cardioverter-defibrillator wires, and peripheral intravascular lines (PIVs) as common triggers for upper extremity DVTs [6]. In the MITH study, patients admitted between 2002 and 2009 who had a CVC placed had a 14-fold higher risk of developing an upper extremity DVT than those who did not [7].

Little information is seen recorded in the current literature with regard to the frequency of conversion of superficial thrombophlebitis of the upper extremity into a DVT. Additionally, there is no mention in the current literature of a butterfly needle used for a routine blood draw provoking a DVT, making our case report a unique addition to this growing body of evidence.

As early as 1856, the etiology of thrombosis was described in a simplified way, describing three factors in what became known as Virchow’s triad. Consisting of stasis in the laminar flow of blood, vessel damage, and a state of hypercoagulability, this thinking helps identify causes of thrombosis and subsequent embolic event while assisting in assessing the risk in patients with some or all of the factors of the triad. Our patient had an instance of vascular damage when he had his blood drawn, though as stated above, the use of a butterfly needle for a blood draw has not been shown to provide significant vascular damage before. There was no evidence of blood flow stasis, such as immobilization, paralysis, or spinal damage, and the patient had never been diagnosed with coagulopathy to suggest hypercoagulability.

Our patient, in particular, was ultimately transitioned from a heparin drip to a three-month course on a direct factor Xa inhibitor. He had an uneventful inpatient course and was eventually discharged to follow up with his primary care for testing to evaluate for a clotting disorder leading to a hypercoagulable state testing. Our patient was treated according to the standard of care with regard to anticoagulation. Thrombolysis is not yet accepted as a standard of care for upper extremity DVT treatment as no randomized control trials have been performed assessing this intervention compared to anticoagulation [8].

## Conclusions

Upper extremity superficial and DVTs are uncommon but becoming more frequently diagnosed conditions as endovascular technology develops and central venous catheters are being utilized more often. Multiple case reports and reviews highlight this emerging condition and describe various etiologies, clinical identifiers, and complications of venous thrombosis. This case report describes a patient without the typical risk factors that had developed a superficial thrombosis secondary to a non-indwelling needle stick, with rapid propagation of the clot to involve the deeper veins of the upper extremity and the neck. This case highlights a unique disease progression after a usually benign procedure and emphasizes the importance of providing patients with strict return precautions to help catch potentially deadly developing pathology.
